# Biosensor Zebrafish Provide New Insights into Potential Health Effects of Environmental Estrogens

**DOI:** 10.1289/ehp.1104433

**Published:** 2012-04-18

**Authors:** Okhyun Lee, Aya Takesono, Masazumi Tada, Charles R. Tyler, Tetsuhiro Kudoh

**Affiliations:** 1 Biosciences, College of Life and Environmental Sciences, University of Exeter, Exeter, United Kingdom; 2 Department of Cell and Developmental Biology, University College London, London, United Kingdom

**Keywords:** biosensor, EDCs, endocrine-disrupting chemicals, ER, estrogen receptor, Gal4, UAS, upstream activation sequence

## Abstract

Background: Environmental estrogens alter hormone signaling in the body that can induce reproductive abnormalities in both humans and wildlife. Available testing systems for estrogens are focused on specific systems such as reproduction. Crucially, however, the potential for significant health impacts of environmental estrogen exposures on a variety of body systems may have been overlooked.

Objective: Our aim was to develop and apply a sensitive transgenic zebrafish model to assess real-time effects of environmental estrogens on signaling mechanisms in a whole body system for use in integrated health assessments.

Methods: We created a novel transgenic biosensor zebrafish containing an estrogen-inducible promoter derived with multiple tandem estrogen responsive elements (EREs) and a Gal4ff-UAS system for enhanced response sensitivity.

Results: Using our novel estrogen-responsive transgenic (TG) zebrafish, we identified target tissues for environmental estrogens; these tissues have very high sensitivity even at environmentally relevant concentrations. Exposure of the TG fish to estrogenic endocrine-disrupting chemicals (EDCs) induced specific expression of green fluorescent protein (GFP) in a wide variety of tissues including the liver, heart, skeletal muscle, otic vesicle, forebrain, lateral line, and ganglions, most of which have not been established previously as targets for estrogens in fish. Furthermore, we found that different EDCs induced GFP expression with different tissue response patterns and time trajectories, suggesting different potential health effects.

Conclusion: We have developed a powerful new model for understanding toxicological effects, mechanisms, and health impacts of environmental estrogens in vertebrates.

Environmental estrogens are endocrine-disrupting chemicals (EDCs) that can alter hormone signaling in the body. Human exposure to these chemicals has been associated with decreases in semen quality/sperm count (Li et al. 2011), increased incidence of breast cancer (Doherty et al. 2010) and testicular germ cell cancer (Chia et al. 2010), and urogenital tract malformation (Fernandez et al. 2012). In a recent study in a National Health and Nutrition Examination Survey (NHANES) study population, Melzer et al. (2010) reported that higher urinary levels of the weak environmental estrogen bisphenol A (BPA), taken into the body via the diet, were associated with heart disease and diabetes. In fish, exposure to environmental estrogens affects reproductive development, causes feminization of males (Lange et al. 2009), and alters sexual behaviors (Van den Belt et al. 2004; Vos et al. 2000). More than 900 chemicals have been identified with endocrine-disrupting activity, of which 200 showed estrogenic effects (Botham and Holmes 2005). Major international programs have been established to screen and test for endocrine-disrupting activity to avoid potential human and environmental health risks associated with their exposure (e.g., Reif et al. 2010). Critically, however, available screening and testing systems for estrogens are focused on specific individual mechanisms (e.g., estrogen receptor–activated cell lines) or *in vivo* studies that assess effects on reproduction only.

The roles of steroid estrogens in reproductive development are well established, and many of these roles are common across the vertebrate species. Estrogens are fundamental in the growth and development of the ovary in females (Devlin and Nagahama 2002; Richards et al. 1976), and they are also required for spermatogenesis in males (Mahato et al. 2001; O’Donnell et al. 2001). In addition, estrogens are known to play key roles in a wide range of other physiological functions, including immune responses, the central nervous system, and normal somatic cell growth (Filby and Tyler 2005; Gustafsson 2003). In mammals, estrogen signaling operates through two different estrogen receptors (ERs), ERα and ERβ, which have different tissue distributions and regulate different estrogen responses (Pettersson and Gustafsson 2001). In fish, there are three ERs, *esr1, esr2a,* and *esr2b* (Filby and Tyler 2005; Ma et al. 2000; Socorro et al. 2000), which also show different patterns of tissue expression (Filby and Tyler 2005; Froehlicher et al. 2009; Hawkins et al. 2000; Kuiper et al. 1998; Menuet et al. 2002; Sun et al. 1999; Tingaud-Sequeira et al. 2004). However, information on the functional distinctions between the ERs in fish is lacking.

Understanding the physiological effects of estrogenic chemicals would be greatly enhanced by *in vivo* models capable of detecting tissue-specific effects of estrogens with high sensitivity. Transgenic (TG) zebrafish have considerable potential for screening and testing EDCs in order to understand their mechanisms of effect and assess their potential health impacts in both animals and humans (Chico et al. 2008; Moss et al. 2009; Raldua and Babin 2009). In the present study, we established an estrogen-responsive transgenic zebrafish to detect estrogenic signaling of natural (endogenous) hormones as well as exposure to exogenous estrogens, including EDCs, in both embryonic and early larval stages in real time. The system contains an estrogen-inducible promoter that is derived from a short stretch of multiple tandem estrogen-responsive elements (EREs) and is devoid of any tissue-specific enhancer/suppressor elements. To enhance the system’s response sensitivity, we used a Gal4ff-UAS (upstream activation sequence) system not previously applied in a fish biosensor system. The TG zebrafish produced are highly responsive to environmental estrogens and identify a wide range of target tissues, most of which have not been reported previously. The TG fish further showed that different EDCs induced different tissue patterns and response time trajectories. Gal4ff-UAS zebrafish thus provide a highly effective system for studying potential health effects of exposure to estrogenic EDCs, as well as a new and enhanced capability for screening and testing of environmental estrogens.

## Materials and Methods

*Fish husbandry and experiments.* All experimental procedures conducted with animals were in accordance with UK Home Office animal procedures [Animal (Scientific Procedures) Act 1986] and followed strict local ethical review guidelines ensuring their humane treatment and with regard to alleviation of suffering.

*Generation of ERE-green fluorescent protein (GFP) transgenic fish.* We used polymerase chain reaction (PCR) and two specific primers (5´-CCAGGTCAGAGTGACCTGAGCTAAAATAACACATTCAGCCAGGTCAGAGTG-3´ and 3´-CTGAATGTGTTATTTTAGCTCAGGTCACTCTGACCTGGCTGA​ATGTGTTAT-5´) for EREs (Sathya et al. 1997) were run using polymerase chain reaction (PCR) to produce a template (in eight cycles: denaturation at 96°C for 1 min, annealing at 60°C for 30 sec, and extension at 72°C for 1 min) from which a series of different numbers of tandemly repeated EREs were generated. From the ladder of tandem EREs generated by PCR, the DNA bands for 3EREs were cut and inserted into *Xho*I and *Xba*I fragment sites of pBluescriptKS+ (Agilent Technologies, Santa Clara, CA, USA). The resulting sequence was combined with a TATA sequence (5´-GGCGTCGACTCTAGAG​GGTATATAATAGATCTGCGATCTA​AGTAAGCTTGG-3´ and 3´-CGCGGGCCCGGCTTTACCAACAGTACCGGAATGCCAAGCTTACTTAGATCG-5´), Gal4ff (amplified by PCR using primers 5´-GCCGGGCCCGCCACCATGAGCTACTGTCTTCT-3´ and 3´-GCGGTACCGATTAGTTACCCGGGAGC-5´) and poly A sequences, and inserted into pBR322 *Tol2* vector (Kawakami et al. 2004; Urasaki et al. 2006). The pBR Tol2-3ERE-Gal4ff plasmid (18 ng/µL) was injected into one-cell-stage embryos for UAS-GFP transgenic zebrafish (Kajita et al. 2010) together with transposase mRNA (36 ng/µL), and the injected embryos were raised to adulthood. Eggs were collected from founders and exposed to 100 ng/L 17α-ethinylestradiol (EE_2_) for 3 days, and the GFP-positive embryos were selected and reared to adulthood. We established three stable ERE-TG zebrafish lines from different pairs of founder fish (3×ERE:Gal4ff and UAS:GFP) that all showed the same GFP expression pattern. We used the F_2_ generation of the ERE-TG zebrafish derived from line one in all studies reported here.

*Zebrafish embryo chemical exposures.* We selected a series of chemicals to assess the responsiveness of our TG zebrafish to estrogens, including the natural steroid estrogen 17β-estradiol (E_2_); EE_2_, which is used in contraceptive pills and hormone replacement therapy; BPA, used widely as a plasticizer; and 4-nonylphenol (NP), used as an industrial surfactant (Goodhead and Tyler 2009). All of these chemicals have been shown to contaminate the aquatic environment and induce feminized responses in fish (Goodhead and Tyler 2009; Tyler et al. 1998). E_2_ (98% purity), EE_2_ (≥ 98% purity), BPA (≥ 99% purity), and NP (Acros Organics) were purchased from Sigma Chemical Co. Ltd. (Poole, UK). Stock chemicals were dissolved in acetone at 10 mg/L for EE_2_ and E_2_, 100 mg/L for BPA, and 500 mg/L for NP; solutions were prepared in glass bottles and stored at 4°C until required. To validate the selectivity of the reporter, we also assessed responses to testosterone (0.1–10/L) and dexamethasone (Dxm; 0.1–10 mg/L), which are structurally similar to estrogens but are not ER ligands. Testosterone and Dxm were dissolved in acetone, as described above for estrogens: the stock concentrations of 1 mg/L and 1 g/L, respectively. The working solutions were prepared 3 days before use, with the required amount of stock solution pipetted into a glass bottle and the solvent evaporated away under a stream of nitrogen. Controls were embryos exposed to culture water alone. The working solutions were made up in embryo culture water and stirred vigorously for 1 day. Beginning 1 hr postfertilization (hpf), TG embryos were exposed to a series of chemical concentrations, including those with environmental relevance. To confirm that estrogenic responses (GFP expression) occurred via an ER-mediated pathway, we co-exposed TG zebrafish embryos to EE_2_ and an estrogen receptor antagonist [ICI 182,780 (ICI); Tocris Bioscience, Bristol, UK]. ICI was dissolved in ethanol at a stock concentration of 50 mg/L, and the working concentration was 10 µg/L. All experiments (50 eggs/treatment) were run in duplicate and were repeated at least five times.

*Analysis of EE_2_ in exposure water.* To assess the response sensitivity of our ERE-TG zebrafish, we measured water EE_2_ concentrations using gas chromatography–mass spectrometry (conducted by Severn Trent Services, Midlands, UK). Nominal test concentrations were highly consistent across the range tested, between 68 and 72% of nominals (specifically, for nominal EE_2_ exposure concentrations of 1, 2.5, and 10 ng/L, measured levels were 0.72, 1.71, and 7.25 ng/L, respectively). Concentrations of EE_2_ in water controls were nondetectable (< 0.05 ng/L).

*Quantification of GFP (EGFP) expression using Western blot analysis.* Four-day-old larvae were dissolved in reduced 2× lithium dodecyl sulfate sample buffer (Invitrogen, Carlsbad, CA, USA) and homogenized using a hand homogenizer. Samples were boiled for 15 min at 70°C, centrifuged, and then analyzed by electrophoresis using NuPAGE NOVEX 4–12% Bis-Tris gel (Invitrogen). Separated proteins were transferred onto a nitrocellulose membrane. The membranes were blocked with 5% milk in 1× phosphate-buffered saline (PBS) + 0.01% Tween (PBSTx) at room temperature for 1 hr and incubated overnight at 4°C with rabbit anti-GFP antibody (1:2,500) (AMS Biotechonology, Abingdon, UK). The membrane was then washed three times (15 min each) with 1× PBSTx at room temperature for 15 min and then incubated with horseradish peroxidase–conjugated goat anti-rabbit IgG (1:2,000) (Invitrogen) at room temperature for 1 hr. The membrane was then washed three times with 1× PBSTx as described above. For detection, we used luminol reagent for Western blotting (Thermo Scientific) and analyzed the intensity of GFP using ImageJ (http://rsbweb.nih.gov/ij/). Results were normalized to the total α-tubulin level and are presented as fold increase in GFP over the level of unexposed embryos.

*Image analysis.* Live larvae were anesthetized with 0.4% tricaine, mounted in 0.7% low-melting agarose in embryo culture medium, and placed onto a glass-bottom 35-mm dish (MatTek, Ashland, MA, USA). Images of stained larvae oriented in lateral, dorsal, and ventral views were obtained using an inverted confocal microscope (Zeiss, Cambridge, UK), with a 10× objective lens. Z-stacks of line-averaged (four lines) sections were obtained by scanning the area of 102.4 µm × 102.4 µm (0.1 µm/pixel) and 6-µm steps over a total vertical distance of 180–240 µm, and were reconstituted using the LSM510 Meta program (Zeiss). Images from parts of the body of larvae were aligned and the contrast was adjusted using Adobe Photoshop 7 (Adobe Systems, San Jose, CA, USA), keeping the same intensity of the adjustment for control and all treated samples. We used fluorescence microscopy (Leica DMI 4000 B; Leica Microsystems Ltd., Milton Keynes, Bucks, UK) to examine tissue-response dynamics and sensitivity for EE_2_ exposure.

*Statistical analysis.* All data are reported as mean ± SE. Statistical comparisons were performed between controls and each exposed group using Student’s *t*-test. Statistical significance is indicated at *p* < 0.05 or *p* < 0.01.

## Results and Discussion

**Figure 1:**
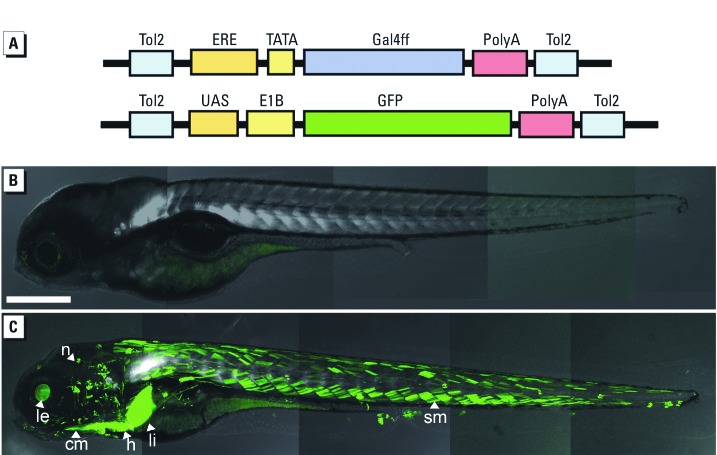
3ERE‑Gal4ff/UAS‑GFP system in zebrafish. (*A*) The ERE-Gal4ff transgene contains three synthetic EREs, one TATA, a Gal4ff reporter, and two *Tol2* elements; and the UAS‑GFP transgene contains five tandem repeats of the UAS-Gal4 binding sequence, an E1B minimal promoter, and an EGFP reporter. An estrogenic signal is detected when the chemical–ER complex binds to the ERE, which activates Gal4ff; subsequently, Gal4ff protein binds to UAS to induce EGFP. (*B*,*C*) Induction of GFP in larvae of ERE-Gal4ff/UAS‑GFP double transgenic fish. Four-day-old larvae were exposed to water alone (control; *B*) or E_2_ (100 ng/L). GFP expression (green) is present in the cranial muscle (cm), heart (h), lens (le), liver (li), neuromast (n), and somite muscle (sm). Bar = 200 µm.

In creating these fish, we synthesized two transgenic vectors that contained ERE-Gal4ff and UAS-GFP (Figure 1). The three tandem repeats of ERE drive the Gal4ff reporter (a modified form of Gal4 transcription factor), providing high transcriptional activation with low toxicity. The Gal4ff transcription factor subsequently binds to UAS and activates GFP, and the sequential activation of the two reporters amplifies the signal. Double TG fish (3×ERE:Gal4ff, UAS:GFP) were generated by injecting ERE-Gal4ff vector into preexisting UAS-GFP TG fish (Kajita et al. 2010) using a *Tol2* transposon system (Kawakami et al. 2004; Urasaki et al. 2006).

We examined the GFP response to EE_2_ in three different ERE-TG zebrafish lines and confirmed the same responses across these lines. Furthermore, the lines have now been maintained over four generations, and the pattern of GFP response has not changed, indicating that the transgene is very stable and that the response is consistent over successive generations without a silencing effect. Furthermore, we confirmed that the ER response to estrogen was specific, because neither testosterone (0.1–10 µg/L) nor Dxm (0.1–10 mg/L), both of which are structurally similar to estrogens but do not bind to ERs, activated the reporter [see Supplemental Material, Figure 1 (http://dx.doi.org/10.1289/ehp.1104433)].

Our examination of GFP expression in control 4-day-old TG fish larvae showed weak basal GFP expression in the otic vesicle, with a 57% frequency, and in the heart, with a lower frequency (40%). The variation in GFP expression in the unexposed larvae may suggest that differences in estrogen levels in fish embryos and larvae occur from an early developmental stage. Whether these differences relate to sex has not been determined because there are no specific sex probes available for this species. It is also possible that maternal loading of estrogens in the yolk might account for the variation in the estrogen signal in the TG fish larvae.

**Figure 2:**
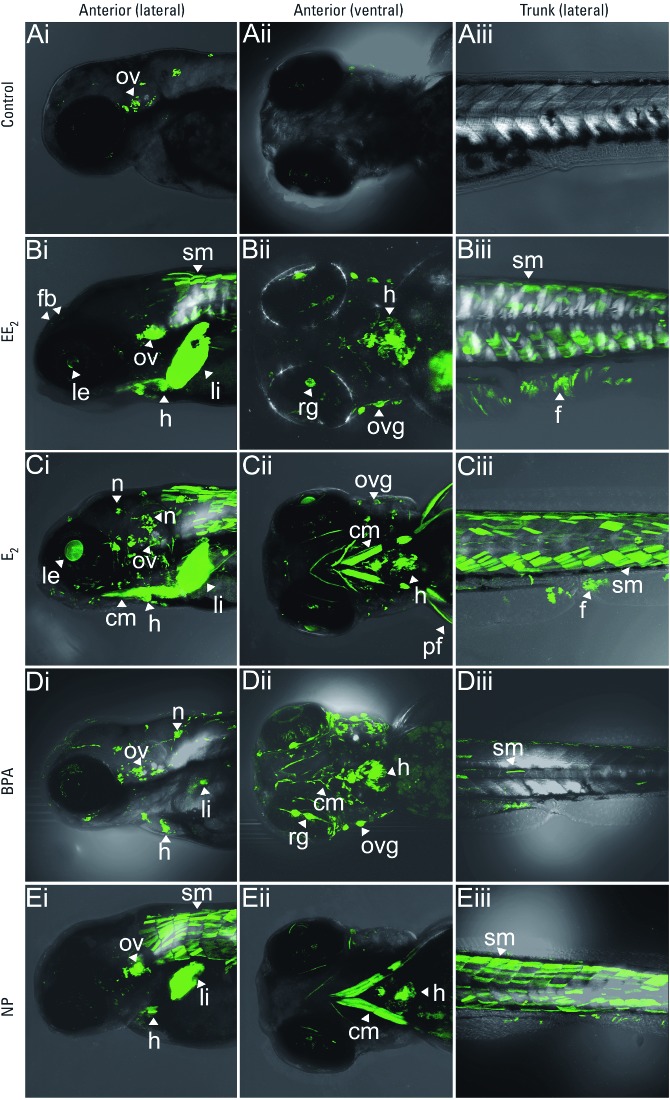
Expression of GFP in transgenic zebrafish larvae exposed to estrogenic chemicals shown in the head with lateral (*i*) and ventral (*ii*) views and in the trunk with lateral view (*iii*). Transgenic zebrafish larvae were exposed to water alone (control; *A*), EE_2 _(100 ng/L; *B*), E_2 _(100 ng/L; *C*), BPA (1 mg/L; *D*), or NP (10 µg/L; *E*) for 4 days. GFP induction was observed in the otic vesicle (ov) in the control larvae (*A*), but the estrogenic chemicals induced different tissue patterns of GFP expression (*B–E*). EE_2_ (*B*) and E_2_ (*C*) induced GFP expression in cranial muscles (cm), fin (f), forebrain (fb), heart (h), lens (le), liver (li), neuromast (n), ov, ov ganglions (ovg), pectoral fin (pf), retinal ganglions (rg), and somite muscles (sm). In BPA-exposed larvae (*D*), h and cm expression was enhanced; in NP-exposed larvae (*E*), strong GFP expression was detected in the cm and sm. Bars = 200 µm.

Exposure to different estrogens induced different tissue patterns of GFP induction (Figure 2). In larvae exposed to EE_2_ (Figure 2B), GFP expression was observed in the liver and forebrain, as has been reported with estrogen-responsive TG GFP fish lines employing a vitellogenin promoter and a *cyp19a1b* promoter, respectively (Bogers et al. 2006; Chen et al. 2010; Legler et al. 1999; Tong et al. 2009). In most estrogen-responsive TG fish (both zebrafish and medaka) systems, responses have largely been restricted to the liver and have a relatively low sensitivity (Bogers et al. 2006; Chen et al. 2010; Legler et al. 1999; Tong et al. 2009). Previously, the most sensitive TG fish showed a response to EE_2_ at 10 ng/L after 30 days of exposure, as determined by luciferase activity (Bogers et al. 2006). In a recently established transgenic zebrafish containing GFP and five consecutive elements upstream of a c-*f*os minimal promoter, E_2_ induced GFP expression in the heart (Gorelick and Halpern 2011). However, these responses were induced by very high exposure concentrations of estrogens (E_2_ at 1–100 µg/L) that are not environmentally relevant. In the TG fish in the present study, we found specific and strong GFP expression in tissues that other biosensor studies have not identified as targets for estrogen, including the heart, skeletal muscle, neuromasts, and otic vesicle/eye and otic vesicle/eye ganglions (Figure 2B,C). In addition, we quantified *in vivo* responses to environmentally relevant doses of estrogens in real time. GFP expression in the heart was confirmed by the periodical contractile movement of the two GFP expressing domains in the artery and in the ventricle, synchronizing with the heartbeat in the live fish. GFP expression in somite muscle was more intense in anterior somites than in posterior somites at early larval stages, but posterior expression increased gradually over time in later larval stages. Each expression domain outlined the shape of a single muscle myotube. Interestingly, with low EE_2_ exposures GFP expression in muscle somites was often observed as a mosaic pattern (it was expressed in only some of the myotubes). At high EE_2_ exposure, GFP expression was present in a uniform manner in the myotubes.

We also observed specific GFP expression in the neuromasts of the lateral line of the head and in ganglions adjacent to the otic vesicles and eyes, which showed typical large masses of neurons and neurite (see Figure 2Ci,Dii for responses to E_2_ and BPA, respectively). Although neuromast-specific responses to estrogens have not been reported in other biosensor fish, *esr2a* and *esr2b* are highly expressed in the neuromasts, and knockdown of *esr2b* abolishes neuromast development, indicating a crucial role of ER signaling for neuromast development/function (Froehlicher et al. 2009; Tingaud-Sequeira et al. 2004). The GFP tissue expression pattern induced by natural steroidal estrogen (E_2_) is similar to that induced by EE_2_ (Figure 2B,C). Studies using real-time PCR have shown that ER transcripts are expressed in the liver, heart, brain, testis, and kidney after exposure to estrogen (Chandrasekar et al. 2010), which is consistent with the GFP responses in our TG fish.

In embryos exposed to BPA, we observed strong GFP expression in the heart, cranial muscle, and otic vesicle and retinal ganglions with long neurites (Figure 2Di,Dii). In contrast to EE_2_ and E_2_ exposures, GFP expression in liver and somite muscle were relatively weak in response to BPA. GFP expression induced by NP was strongest in the liver and somite muscle but was less intense in the otic vesicle and heart (Figure 2E). These expression patterns suggest that different estrogens have common target tissues (e.g., liver, heart, muscle, otic vesicle), but they have different degrees of effect on the different tissues (i.e., tissue-specific responsiveness), with different potential health effect outcomes.

**Figure 3:**
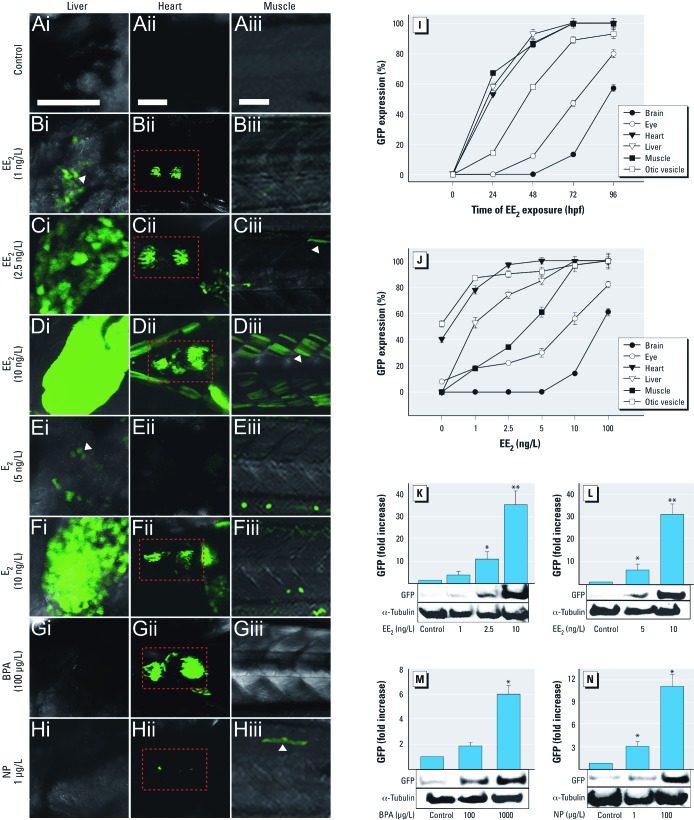
GFP expression in transgenic zebrafish embryos. GFP expression in control (unexposed) embryos (*A*) or in embryos exposed to EE_2_ (1, 2.5, and 10 ng/L; *B–D*), E_2_ (5 and 10 ng/L; *E,F*), BPA (100 µg/L; *G*) or NP (1 µg/L; *H*). Bars = 100 µm. Images focus on liver (lateral view; *i*), heart (ventral view; *ii*), and somite muscle (lateral view; *iii*). GFP expression was detected in the liver after exposure to EE_2_ at 1 ng/L (*Bi*) and in liver, heart, and somite muscle after exposure to EE_2_ at 2.5 and 10 ng/L (*C,D*). After E_2_ exposure, a few GFP-expressing cells were observed in the liver at 5 ng/L (*Ei*), whereas strong GFP expression was detected in the liver and heart after exposure to E_2_ at 10 ng/L (*F*). After BPA exposure, GFP was preferentially expressed in the heart (*Gii*), but not in the liver or somite muscle (*Gi*,*Giii*). Weak GFP expression was detected in the heart and somite muscle after NP exposure (*Hii,Hiii*). Red boxes indicate GFP expression in the heart. Time-related (*I*) and concentration-dependent (*J*) analyses of GFP expression performed 4 days after EE_2_ exposure. (*K–N*) Dose response of GFP induction by EE_2_ (*K*), E_2_ (*L*), BPA (*M*), and NP (*N*) measured by Western blotting. α‑Tubulin was used as a loading control; analyses were conducted three times, and data are reported as mean ± SE. *p < 0.05, and **p < 0.01 compared with control.

To understand stage-dependent tissue responses to estrogen, we examined the response dynamics of different tissues after exposure to EE_2_ (100 ng/L) from 1 hpf to 96 hpf (Figure 3I). We observed GFP expression at 24 hpf in the liver, heart, and muscle; expression increased progressively until 96 hpf, when it reached a maximal expression level. In contrast, GFP expression in the brain and nervous system appeared later in development; GFP was seen in the otic vesicle only after 48 hpf, and in the eye and forebrain after 72 hpf (Figure 3I). Data indicate that estrogenic exposure affects different tissues in a life-stage–dependent manner, and illustrate the usefulness of the ERE-TG fish model in investigating time-related effects of estrogens in an intact animal.

ERE-TG fish embryos were exposed to various concentrations of the selected EDCs (Figure 3) to determine whether they were capable of detecting responses for exposures to environmentally relevant concentrations. Responses to the most potent estrogen, EE_2_, were titrated down to 1 ng/L (measured concentration, 0.72 ng/L), where a low-level GFP response was induced in cells in the liver (Figure 3Bi). Higher exposure concentrations resulted in progressively higher GFP expression levels, and a nominal concentration of EE_2_ (10 ng/L; measured concentration, 7.25 ng/L) induced considerable expression throughout the liver; this was accompanied by GFP expression in the muscle (somite and cranial muscle) and heart (Figure 3D). Concentration-dependent GFP expression was further confirmed quantitatively via Western blot analyses. The level of GFP expression increased dramatically and in a concentration-dependent manner for both EE_2_ (1–10 ng/L; Figure 3B–D,J,K) and E_2_ (5–10 ng/L) (Figure 3E,F,L). The threshold concentrations for NP and BPA (1 µg/L and 100 µg/L, respectively) were significantly higher, showing a low relative potency of these environmental estrogens compared to the steroidal estrogens EE_2_ and E_2_ (Figure 3G,H,M,N). Both BPA and NP also bind to other soluble receptors, such as the androgen receptor and the thyroid hormone receptor; these activities could influence pathways that intersect with the ER pathway.

**Figure 4:**
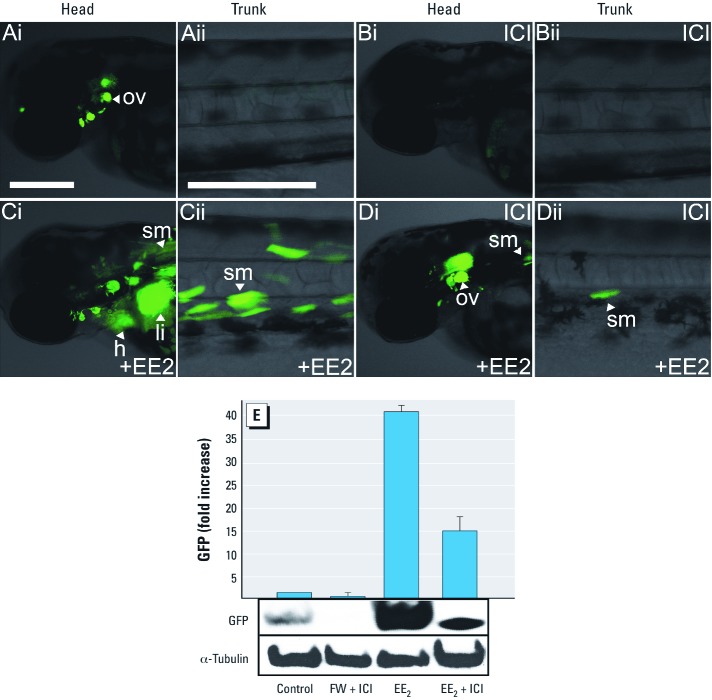
Effect of the ER inhibitor ICI on suppression of GFP expression in ERE-TG zebrafish. ERE‑TG embryos were exposed to (*A*) water alone (control), (*B*)**ICI alone (10 µg/L), (*C*)**EE_2_ alone (20 ng/L), or (*D*) ICI plus EE_2_ as a mixture. Bars = 200 µm. ICI alone suppressed GFP expression in the otic vesicle of fish (*Bi*). Strong GFP expression was present after EE_2_ exposure (*C*), whereas GFP expression was suppressed after treatment with ICI plus EE_2_ as a mixture (*D*). (*E*) Western blotting further confirmed that GFP expression was significantly suppressed by ICI in a quantitative manner.

To examine whether estrogen-induced GFP expression in ERE-TG fish was mediated via ERs, we conducted additional experiments to specifically suppress ER activity in the ERE-TG zebrafish by adding ICI to the embryo culture. Exposure of ERE-TG zebrafish larvae to ICI (Figure 4Bi) abolished GFP expression observed in the otic vesicles of nonexposed fish (Figure 4Ai). Moreover, exposure to EE_2_ plus ICI (Figure 4Di,Dii) greatly reduced GFP expression in all of the tissues (i.e., liver, heart, muscle, neuromasts, ganglions) compared with EE_2_ alone (Figure 4Ci,Cii). Consistent with the fluorescent imaging data, Western blot analysis confirmed that ICI treatment led to drastic inhibition of both basal GFP expression in control fish and GFP expression in EE_2_-treated fish (Figure 4E). These data suggest that a major part of GFP response in these TG fish is mediated by the ERs.

The functional consequences of environmental estrogen exposures—especially during early life—are uncertain, and information concerning the molecular mechanisms underlying estrogen-mediated physiological and pathological consequences of these exposures is limited. Transgenic zebrafish and medaka (*Oryzias latipes*) have been developed previously to detect exposure to environmental estrogens, but expression of the reporter genes in these fish has been restricted to the liver and gonads, most likely because the gene-specific promoters used (e.g., vitellogenin) are predominantly active in these tissues (Legler et al. 1999; Salam et al. 2008; Zeng et al. 2005). The system described here employs a synthetic ERE that lacks any additional enhancer/suppressor elements that could bias the estrogen response or restrict it to specific tissues. Therefore, our model can detect estrogenic responses in a wider range of tissues than other available TG fish systems.

Our modified reporter system considerably enhances sensitivity in a variety of tissues, allowing us to detect responses to chemical exposures at environmentally relevant concentrations. Although we identified several novel target tissues for estrogens, we used only one type of ERE and surrounding sequence, which will not be effective for all estrogenic signaling systems. Specifically, ERs that work through Sp1 or AP1 sites would not be detected by this assay, nor would EREs with half-binding sites only.

Specific cell types in liver, gonad, and forebrain are responsive to estrogen (Chen et al. 2010; Hano et al. 2007; Legler et al. 1999; Tong et al. 2009), and neuromast cells have elevated expression levels of *esr2* genes (Froehlicher et al. 2009). In addition to these target tissues, we demonstrate for the first time that muscle (somite and cranial), heart, and many ganglion cells also respond to estrogen exposure, thus activating the estrogen signaling cascade in these cells. E_2_ caused a significant increase in expression of *esr1* and *esr2* in cardiac myocytes, in which estrogen regulates expression of specific cardiac genes (Kahlert et al. 1997). Furthermore, exposure to the environmental estrogen BPA has been reported to result in heart defects (Melzer et al. 2010). These data, together with our finding of specific responses of GFP to estrogens, suggest a crucial role of estrogenic signaling in the developing heart. Identifying target genes of the estrogenic signaling cascade in these tissues, as well as detailed analyses of the processes affected, will help in understanding the functional significance of these responses. Despite the fact that three ERs are known to be expressed widely in the embryo and larvae of fish (Froehlicher et al. 2009; Tingaud-Sequeira et al. 2004), we detected estrogenic GFP responses in our TG fish only in a limited number of tissues (e.g., heart, muscle, brain); this may indicate that estrogen signaling requires tissue-specific cofactors or coreceptors to activate the estrogen cascade and/or that signaling operates through the membrane-associated ER GPR30 (G protein-coupled receptor 30) in some tissues (Filardo et al. 2007; Revankar et al. 2005). Tissue-specific estrogenic responses differed among the environmental estrogenic chemicals we tested. For instance, BPA caused a strong response in the heart, whereas NP caused a more potent response in skeletal muscle. These findings suggest that although these chemicals are both classified as environmental estrogens, they may have different effects on health. Future investigations of tissue-specific responses should help determine whether this is indeed the case.

Our TG system effectively detected responses to environmental estrogens (NP and BPA) of a comparatively weak potency compared with steroidal estrogens, and tissue response patterns differed for the different estrogens tested. This suggests that the system has considerable potential for screening and characterizing estrogenic properties of chemicals and that it offers a new model for understanding effect mechanisms. Applied to embryos/larval stages, this system could be developed as a high throughput screening system, similar to zebrafish embryo systems used to test drugs in the pharmaceutical industry (Chakraborty et al. 2009; Xu et al. 2010).

We have developed a powerful model system that can be used to screen and test environmental estrogens, allowing for targeting of tissue-specific studies to identify molecular mechanisms that underlie estrogen signaling pathways, and for understanding the physiological and pathological impacts of these compounds.

## Supplemental Material

(4.6 MB) PDF
