# Supplementary material for: Biosensor Zebrafish Provide New Insights into Potential Health Effects of Environmental Estrogens
Source: Environ Health Perspect. 2012 Apr 18;120(7):990–6. doi: 10.1289/ehp.1104433 (PMC3404660; doi:10.1289/ehp.1104433)
Supplement: (4.6 MB) PDF [file ehp.1104433.s001.pdf]

Supplemental Material

Biosensor Zebrafish Provide New Insights into Potential Health Effects of Environmental Estrogens

Okhyun Lee[1], Aya Takesono[1], Masazumi Tada[2], Charles R. Tyler[1]* and Tetsuhiro Kudoh[1]

[1] Biosciences, College of Life and Environmental Sciences, University of Exeter, Exeter, U.K.

[2] Department of Cell and Developmental Biology, University College London, London, UK

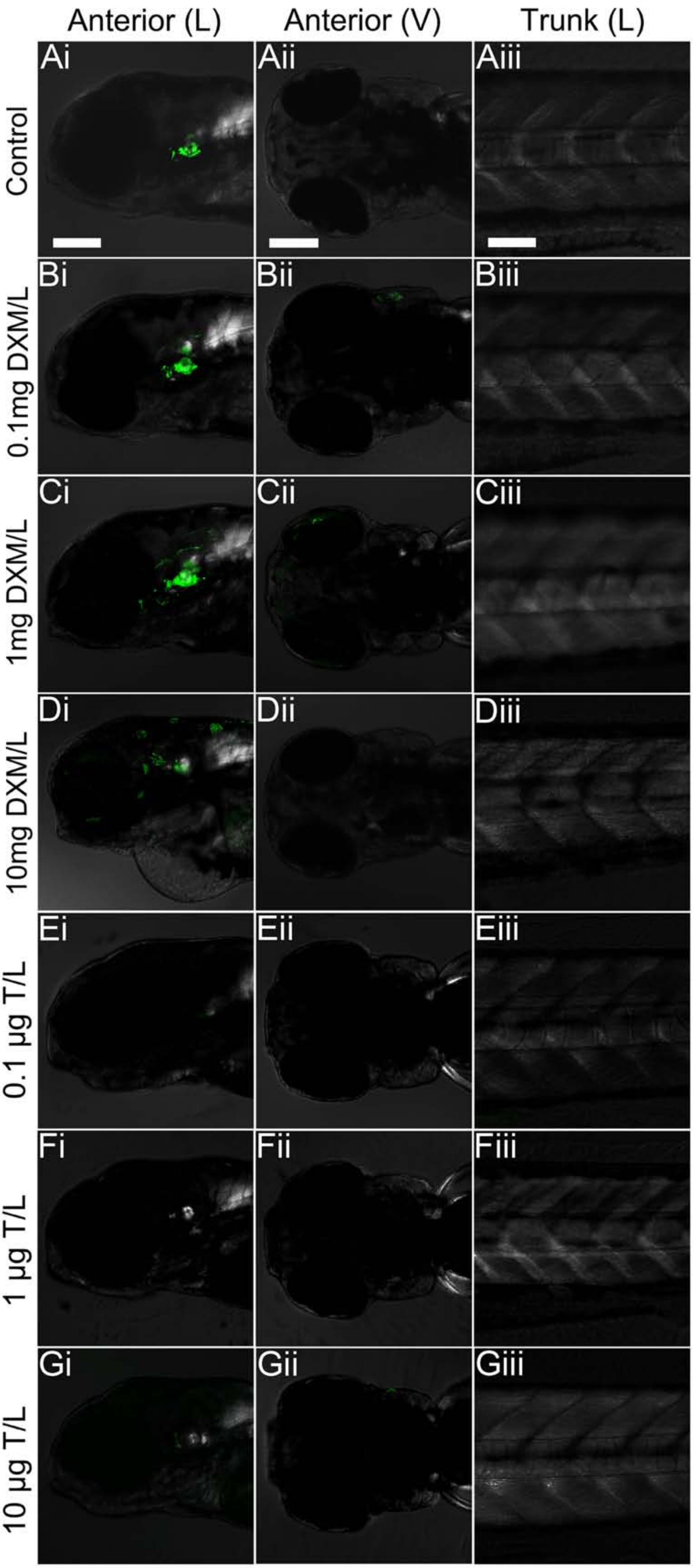

Anterior (L)
Anterior (V)
Trunk (L)
Control
0.1mg DXM/L
1mg DXM/L
10mg DXM/L
0.1 µg T/L
1 µg T/L
10 µg T/L
Ai
Aii
Aiii
Bi
Bii
Biii
Ci
Cii
Ciii
Di
Dii
Diii
Ei
Eii
Eiii
Fi
Fii
Fiii
Gi
Gii
Giii

Supplemental figure 1 legend

Figure 1. Expression of GFP in zebrafish larvae exposed for 4 days to the chemicals dexamethasone (dxm) and testosterone (T) that are structurally similar to estrogens but do not bind to ERs. Unexposed controls (A), Dxm exposed (0.1, 1 and 10 mg/L) (B-D) and T exposed (0.1, 1 and 10 µg/L) (E-G). Fluorescence was detected using an inverted confocal microscope (Zeiss). Head with lateral (L) and ventral (V) views (i and ii) and trunk (iii) with lateral view. GFP was observed in the otic vesicle (ov) in both the unexposed (control) and exposed (dxm) larvae, but there was no GFP activation for any of the tissues seen for the exposures to estrogens. These findings were consistent in duplicate treatments and across two separate assays. Scale bars: 100 µm
